# Association of circulating ANGPTL 3, 4, and 8 levels with medical status in a population undergoing routine medical checkups: A cross-sectional study

**DOI:** 10.1371/journal.pone.0193731

**Published:** 2018-03-14

**Authors:** Jun Morinaga, Jiabin Zhao, Motoyoshi Endo, Tsuyoshi Kadomatsu, Keishi Miyata, Taichi Sugizaki, Yusuke Okadome, Zhe Tian, Haruki Horiguchi, Kazuya Miyashita, Nobuhiro Maruyama, Masashi Mukoyama, Yuichi Oike

**Affiliations:** 1 Department of Molecular Genetics, Graduate School of Medical Sciences, Kumamoto University, 1-1-1 Honjo, Chuo-ku, Kumamoto, Japan; 2 Department of Nephrology, Graduate School of Medical Sciences, Kumamoto University, 1-1-1 Honjo, Chuo-ku, Kumamoto, Japan; 3 Department of Clinical Investigation, Kumamoto University Hospital, 1-1-1 Honjo, Chuo-ku, Kumamoto, Japan; 4 Immuno-Biological Laboratories Co., Ltd., Fujioka, Gunma, Japan; The University of Tokyo, JAPAN

## Abstract

**Purpose:**

Angiopoietin-like proteins (ANGPTLs) 3, 4, and 8 reportedly contribute to progression of metabolic disease, a risk factor for cardiovascular disease (CVD). The purpose of this study was to investigate whether circulating ANGPTL levels are associated with CVD risk after adjustment for potential confounding factors.

**Methods:**

We conducted a single center, cross-sectional study of 988 Japanese subjects undergoing routine health checks. Serum ANGPTL3, 4, and 8 levels were measured using an enzyme-linked immunosorbent assay. Using multiple regression analysis we evaluated potential association of circulating ANGPTL3, 4, and 8 levels with general medical status including age, sex, smoking, drinking, obesity, hypertension, impaired glycometabolism, dyslipidemia, hyperuricemia, hepatic impairment, chronic kidney disease, anemia, cardiac abnormality, and inflammation.

**Results:**

Circulating ANGPTL3 levels were relatively high in health-related categories of hepatic impairment and inflammation. Circulating ANGPTL4 levels were also significantly high in impaired glycometabolism or hepatic impairment but decreased in inflammation. Finally, increased ANGPTL8 levels were observed in obesity, impaired glycometabolism and dyslipidemia. Particularly, increased levels of circulating ANGPTL8 were positively correlated with circulating triglycerides and LDL-cholesterol levels and inversely correlated with circulating HDL-cholesterol levels.

**Conclusions:**

Circulating ANGPTL3, 4, and 8 levels reflect some risk factors for CVD development.

## Introduction

Cardiovascular disease (CVD) is a serious health concern worldwide [1, 2]. To prevent CVD development, early diagnosis of risk factors, including hypertension, diabetes, and dyslipidemia, is important in order to devise timely therapeutic interventions [1, 3]. Relevant to treatment, development of statins as inhibitors of hydroxymethylglutaryl-CoA reductase aimed at reducing plasma low-density lipoprotein (LDL)-cholesterol levels has decreased the number of CVD events, as atherosclerosis due to ectopic accumulation of cholesterol in vessel walls underlies CVD pathology [4, 5]. Clinical studies confirm that lowering plasma LDL-cholesterol concentrations is superior to interventions targeting circulating triglyceride (TG) or high-density lipoprotein (HDL)-cholesterol levels in protecting against CVD development [6, 7]. Therefore, it is considered critical in some cases to more effectively lower plasma LDL-cholesterol concentrations by supplementing statins with medications such as ezetimibe or antibodies against proprotein convertase subtilisin/kexin type (PCSK) 9 as primary and secondary prevention against CVD development. Development of novel drugs reducing plasma LDL cholesterol levels is also an ongoing goal.

Angiopoietin-like proteins (ANGPTLs) are a family of secreted factors structurally similar to angiopoietin and are characterized by an N-terminal coiled-coil domain and a C-terminal fibrinogen-like domain, except for ANGPTL8, which lacks the C-terminal fibrinogen-like domain [8, 9]. Among eight ANGPTLs, ANGPTL3, 4, and 8 exhibit a sequence that binds to lipoprotein lipase (LPL), an enzyme that hydrolyzes TG circulating in capillaries of adipose tissues and muscle [8–10]. Several reports indicate that ANGPTLs 3, 4, and 8 antagonize LPL activity, resulting in increasing plasma TG concentrations [9, 11] However, some reports show that loss-of-function mutations in human *ANGPTL3* and *ANGPTL4* decrease circulating TG concentrations, whereas loss-of-function mutations in human *ANGPTL8* have no significant effect on circulating TG levels [9, 12, 13]. Interestingly, loss-of-function mutations in *ANGPTL4* in humans are associated with increased levels of high-density lipoprotein (HDL)-cholesterol, whereas comparable mutations in human *ANGPTL3* and *ANGPTL8* are associated with decreased circulating HDL-cholesterol levels [9, 12, 13]. Moreover, loss-of-function mutations in human *ANGPTL3* and *ANGPTL8* are associated with decreased circulating LDL-cholesterol levels, whereas comparable mutations in human *ANGPTL4* have no significant effect on those levels [9, 12, 13]. These findings suggest overall that ANGPTLs 3, 4, and 8 play different roles in lipid metabolism.

Recent studies have demonstrated that ANGPTL3 suppression by antibody or antisense oligonucleotides represents a new therapeutic strategy to reduce plasma LDL cholesterol and TG levels for patients with dyslipidemia [12, 14, 15]. This approach has received much attention, as patients undergoing treatment with other lipid-lowering drugs have achieved greater reductions in LDL-cholesterol levels when an ANGPTL3-suppressing drug was added to the treatment regime.

Given that ANGPTL3, 4, and 8 proteins are secreted, their concentrations in circulation are reportedly associated with CVD risk factors such as dyslipidemia and impaired glycometabolism, although these associations vary between studies [16–21]. However, there has been little characterization of potential association of ANGPTL3, 4, and 8 with overall human medical status (including CVD risk) in a single population. Furthermore, although some ANGPTL proteins, among them ANGPTL3 and 4, are proteolytically cleaved in the linker region between the coiled-coil domain and fibrinogen like domain and circulate as both full length and truncated forms, previous reports do not always specify which ANGPTL form is under investigation [22, 23].

The aim of this study was to analyze circulating levels of ANGPTL3, 4, and 8 and correlate them with metabolic CVD risk factors, making adjustments for confounding factors. In this study, we evaluated total levels of full-length ANGPTL3, full-length plus coiled-coil domain of ANGPTL4, and full-length ANGPTL8 in circulation from a population of patients undergoing routine medical checkups.

## Materials and methods

### Subjects

This study was conducted using an observational, cross-sectional design targeting a population undergoing health check-ups at a single Japanese center. Study participants were analyzed in fasting conditions. In 2009, a total of 998 subjects were recruited to a health examination center of the Japanese Red Cross Kumamoto Hospital. After exclusion of 10 subjects who did not consent to study participation, we measured serum ANGPTL3, 4, and 8 levels in 988 subjects. Of those 988 subjects, 188 showed missing laboratory tests, including 175 subjects missing Hemoglobin A_1c_ (HbA_1c_) values, 5 lacking creatinine, and 188 lacking high-sensitivity C reactive protein (hs-CRP) values, and were excluded, leaving 800 subjects. This study was conducted in with keeping Helsinki Declaration and with approval of ethics committees for clinical research at Kumamoto University. Written informed consent was obtained from all participants.

### Clinical evaluation and laboratory testing

Smoking habits were defined as current smoking status. A drinking habit was defined as daily alcohol intake ≥ three times a week. Obesity was defined as a body mass index (BMI) ≥25 kg/m^2^. Hypertension was defined as past history, current use of anti-hypertensive agents, systolic blood pressure (SBP) ≥140 mmHg, or diastolic blood pressure (DBP) ≥90 mmHg. Impaired glycometabolism was defined as past history or history of diabetes, current use of insulin or glucose-lowering agents, fasting plasma glucose ≥126 mg/dl (≥7.0 mmol/l), or HbA1_C_ (Japanese Diabetic Society value) ≥6.1%. Dyslipidemia was defined as past history, current use of lipid lowering agents, TG ≥150 mg/dl, LDL cholesterol ≥140 mg/dl, or HDL cholesterol <40 mg/dl. Hyperuricemia was defined as past history, or uric acid >7.0 mg/dl. Hepatic impairment was defined as past history of liver disease such as viral hepatitis, aspartate transaminase (AST) >40 IU/L, alanine transaminase (ALT) >40 IU/L, or elevation of gamma-glutamyltransferase (GGT) (Male: GGT >70IU/L, Female: GGT>30IU/L). Chronic kidney disease (CKD) was defined as past history of proteinuria, hematuria, glomerulonephritis, or nephrotic syndrome, or estimated GFR <60 ml·min^-1^·1.73m^-2^. Anemia was defined as past history or decreased hemoglobin levels (Male: <13 g/dl, Female: <12 g/dl). Cardiac abnormality was defined as past history of myocardial infarction or electrocardiogram (ECG) abnormality. Inflammation was defined as hs-CRP ≥0.4 mg/dl. All laboratory data except for ANGPTL3, ANGPTL4, and ANGPTL8 levels was gathered in 2009 at the health examination center of the Japanese Red Cross Kumamoto Hospital.

### Measurement of ANGPTLs 3 and 4

Serum specimens were stored at -80°C and thawed twice before assays reported here. In 2017, ANGPTL3, ANGPTL4 and ANGPTL8 protein levels were measured at the Department of Molecular Genetics, Kumamoto University, using a human ANGPTL3 enzyme-linked immune-sorbent assay (ELISA) kit designed to detect full length ANGPTL3 using antibodies respectively targeting N- and C-termini of the protein [Immuno-Biological Laboratories (IBL), Gunma, Japan], and a human ANGPTL4 ELISA kit designed to detect both full length and cleaved protein using antibodies against the N-terminal of ANGPTL4 (IBL). Antibody specificity was confirmed, and none cross-reacted with other ANGPTLs. In particular, antibodies supplied in ANGPTL3 and 4 kits showed little (<0.1%) cross-reactivity with ANGPTL8.

### Development of an ELISA to detect human ANGPTL8

We developed a sandwich ELISA to detect human ANGPTL8 using two antibodies: a rabbit polyclonal antibody (PoAb-169) and a mouse monoclonal antibody (MoAb-21C1). PoAb-169 was developed using a synthesized peptide (169-191aa) of human ANGPTL8, and MoAb-21C1 was developed using recombinant human ANGPTL8. PoAb-169 served as the capture antibody and was used to precoat a 96-well microtiter plate. MoAb-21C conjugated to horseradish peroxidase-conjugated (HRP) served as the detection antibody. Recombinant human ANGPTL8 served as the standard, and human serum samples were prepared as test samples. Specificity of antibodies was confirmed, and none cross-reacted with ANGPTL3 (≤0.1%) or ANGPTL4 (≤0.1%).

### Measurement of serum ANGPTL8

Serially-diluted standards and twenty-fold diluted test samples were added to antibody-precoated plates in duplicate and incubated 60 minutes at 37°C. Plates were then washed with PBS containing 0.05% Tween20 (PBST), and HRP-conjugated MoAb-21C1 mouse IgG Fab’ was added and incubated 30 minutes at 4°C. Plates were washed with PBST, and then tetramethylbenzidine (TMB) solution was added for 30 minutes at room temperature. An equal amount of 1 N H_2_SO_4_ was added to stop the reaction, and absorbance at 450 nm was measured. Human ANGPTL8 levels in test samples were calculated based on evaluation of recombinant human ANGPTL8 standards.

### Statistical analysis

ANGPTL3, ANGPTL4, ANGPTL8, HbA1c, Glucose, TG, AST, ALT, GGT, and hs-CRP were transformed to natural-log values for statistical analysis, as distributions of these variables were skewed. A generalized linear model was used in multiple regression. All statistical analysis was performed using JMP Pro 13.0.0 software (SAS Institute, Cary, NC). All P values were two-tailed, and P<0.05 was taken as statistically significant.

## Results

**Fig 1:**
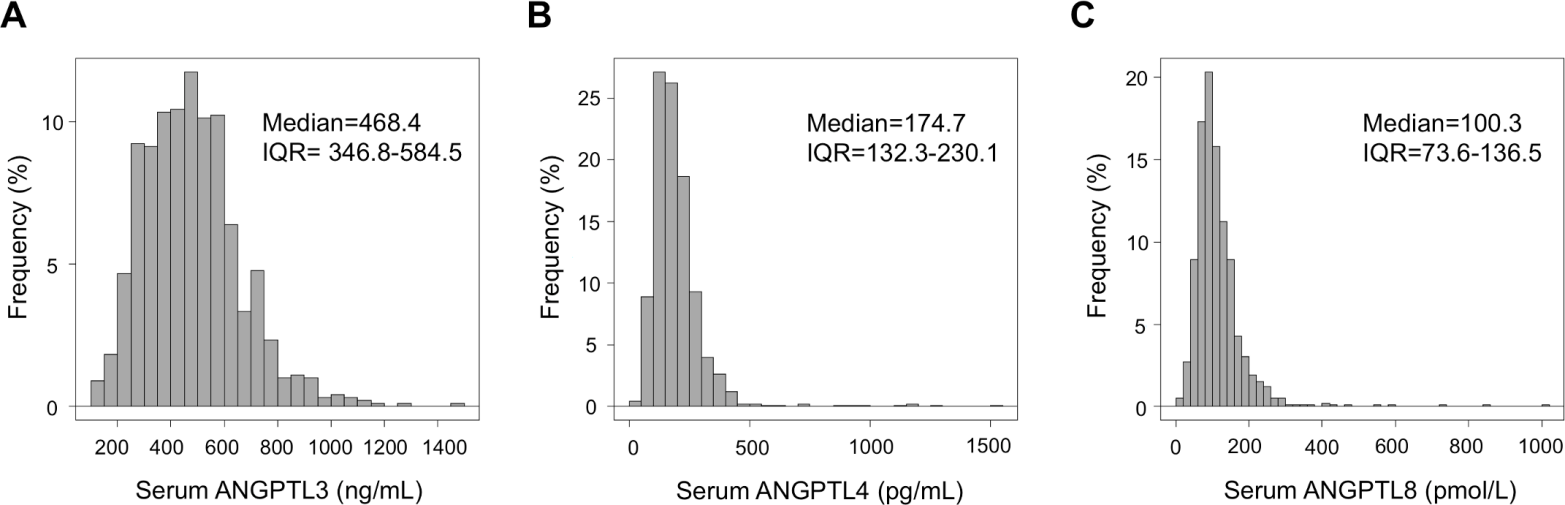
Distribution of circulating levels of (A) ANGPTL3, (B) ANGPTL4, and (C) ANGPTL8. IQR, Interquartile range (n = 988).

Tables 1 and 2 show characteristics of study participants. Distribution of circulating ANGPTL3, 4, and 8 levels is shown in Fig 1. The median ANGPTL3 level was 468.4 ng/mL [inter quartile range (IQR), 346.8–584.5 ng/mL] (Fig 1*A*), the median ANGPTL4 level was 174.7 pg/mL (IQR, 132.3–230.1 pg/mL) (Fig 1*B*), and the median ANGPTL8 level was 100.3 pmol/L (IQR, 73.6–136.5 pmol/L) (Fig 1*C*). We next asked whether these ANGPTL levels were correlated with each other, as all three are linked to changes in lipid metabolism, in spite of verification of no reaction of each antibody in ELISA with other ANGPTLs. However, we observed little correlation of ANGPTL3, 4 and 8 levels in subjects' sera [ANGPTL3 and 4: Spearman’s correlation coefficient (ρ) = -0.029, P = 0.37, ANGPTL3 and 8: ρ = -0.105, P = 0.001, ANGPTL4 and 8: ρ = -0.066, P = 0.038], suggesting that circulating levels of these three ANGPTLs may have different relationships with particular medical issues.

**Table 1:** Baseline characteristics of subjects of study population. Data is shown as the percentage or 95% confidence interval (95%CI). CKD, chronic kidney disease.

Number of subjects	Covariates	Percentage	95%CI
988	Male gender	53.6	(50.5, 56.7)
988	Smoking	18.7	(16.4, 21.3)
988	Drinking (≥3 times weekly)	40.0	(37.1, 43.2)
988	Obesity	27.4	(24.7, 30.3)
988	Hypertension	24.9	(22.3, 27.7)
823	Impaired glycometabolism	7.5	(5.9, 9.5)
988	Dyslipidemia	38.7	(35.7, 41.7)
988	Hyperuricemia	14.5	(12.4, 16.8)
988	Hepatic impairment	23.7	(21.1, 26.4)
983	CKD	8.7	(7.1, 10.7)
988	Anemia	24.1	(21.6, 27.0)
988	Cardiac abnormality	1.1	(0.6, 2.0)
800	Inflammation	4.9	(3.6, 6.6)

**Table 2:** Baseline characteristics of study population subjects. Data is shown as the median or interquartile range (IQR); BMI, body mass index; SBP, systolic blood pressure; DBP, diastolic blood pressure; HbA_1C_, hemoglobin A_1C_; LDL, low-density lipoprotein; HDL, high-density lipoprotein; AST, aspartate transaminase; ALT, alanine transaminase; GGT, gamma-glutamyltransferase; eGFR, estimated glomerular filtration rate; Hb, hemoglobin; and hs-CRP, high-sensitivity C reactive protein.

Number of subjects	Covariates	Median	IQR
988	Age (years)	49	(42, 55.8)
988	BMI (kg/m^2^)	22.8	(21, 25.2)
988	SBP (mmHg)	119	(107, 129)
988	DBP (mmHg)	73	(65, 82)
988	Glucose (mg/dl)	97	(91, 103)
813	HbA_1C_ (%)	5.2	(5.0, 5.4)
988	LDL cholesterol (mg/dl)	119	(101, 140)
988	HDL cholesterol (mg/dl)	64	(53, 77)
988	TG (mg/dl)	95	(66.3, 140)
988	Uric acid (mg/dl)	5.3	(4.3, 6.3)
988	AST (IU/L)	22	(19, 26)
988	ALT (IU/L)	20	(15, 29)
988	GGT (IU/L)	25	(16, 44)
983	Creatinine (mg/dl)	0.73	(0.62, 0.86)
983	eGFR (ml·min^-1^·1.73m^-2^)	78.2	(70.4, 88.0)
988	Hb (g/dl)	14.2	(13.2, 15.2)
800	hs-CRP (mg/dl)	0.04	(0.02, 0.09)

Next, to evaluate potential association of circulating ANGPTLs levels with health-related categories, we conducted multiple regression analysis. Calculation of regression coefficients (β) and P values revealed that circulating ANGPTL3 levels are relatively high in categories such as aging, hepatic impairment, or inflammation (Table 3). By contrast, circulating ANGPTL3 levels were lower in males relative to females (Table 3). Circulating ANGPTL4 levels were relatively high in males and in individuals showing impaired glycometabolism or hepatic function, while ANGPTL4 concentration was relatively low in the aging or in inflammatory conditions (Table 3). Circulating ANGPTL8 levels were relatively high in the aging and in males, the obese, and in dyslipidemia, while ANGPTL8 concentration was relatively low in conditions of impaired glycometabolism (Table 3).

**Table 3:** Association between circulating ANGPTL levels and general medical status (n = 800). A generalized linear model was used. All variables listed were included in the model. ANGPTL, Angiopoietin-like protein; β, regression coefficient; 95% CI, 95% confidence interval; P, probability; and CKD, chronic kidney disease.

	log(ANGPTL3)			log(ANGPTL4)			log(ANGPTL8)		
	β	95%CI	P	β	95%CI	P	β	95%CI	P
Age									
>53	0.066	(0.028, 0.104)	0.001	-0.087	(-0.128, -0.046)	<.001	0.051	(0.003, 0.098)	0.038
45–53	0.000	(-0.036, 0.036)	0.984	0.022	(-0.016, 0.061)	0.260	-0.022	(-0.067, 0.023)	0.336
≤44	-	-	-	-	-	-	-	-	-
Male gender	-0.095	(-0.130, -0.059)	<.001	0.148	0.110, 0.186)	<.001	0.050	(0.006, 0.094)	0.027
Smoking	0.004	(-0.032, 0.040)	0.817	0.022	(-0.018, 0.059)	0.298	-0.023	(-0.069, 0.022)	0.306
Drinking	-0.012	(-0.040, 0.017)	0.410	0.002	(-0.028, 0.033)	0.869	-0.032	(-0.069, 0.003)	0.076
Obesity	-0.024	(-0.055, 0.008)	0.140	-0.002	(-0.036, 0.032)	0.915	0.088	(0.049, 0.128)	<.001
Impaired glycometabolism	0.051	(-0.005, 0.106)	0.072	0.101	0.041, 0.160)	<.001	-0.129	(-0.198, -0.059)	<.001
Hypertension	-0.016	(-0.048, 0.017)	0.350	0.000	(-0.035, 0.035)	0.994	0.009	(-0.032, 0.050)	0.653
Dyslipidemia	-0.020	(-0.048, 0.008)	0.162	0.029	(-0.002, 0.059)	0.063	0.085	(0.050, 0.121)	<.001
Hyperuricemia	0.006	(-0.034, 0.046)	0.760	0.018	(-0.025, 0.061)	0.408	0.044	(-0.007, 0.094)	0.090
Hepatic impairment	0.032	(0.001, 0.063)	0.040	0.037	0.004, 0.070)	0.028	0.029	(-0.010, 0.067)	0.146
CKD	0.001	(-0.037, 0.058)	0.672	0.026	(-0.024, 0.077)	0.308	0.029	(-0.031, 0.089)	0.343
Anemia	0.013	(-0.022, 0.048)	0.478	-0.016	(-0.055, 0.022)	0.401	0.004	(-0.040, 0.049)	0.850
Cardiac abnormality	0.034	(-0.081, 0.149)	0.560	-0.084	(-0.207, 0.040)	0.185	0.039	(-0.105, 0.183)	0.596
Inflammation	0.077	(0.018, 0.136)	0.011	-0.110	(-0.173, -0.046)	<.001	-0.025	(-0.099, 0.049)	0.512

We next asked examined potential associations between circulating ANGPTLs levels and clinical variables shown to be statistically significant in Table 3. Circulating ANGPTL3 levels were positively associated with hs-CRP in the inflammation category, and with AST and GGT in the category of hepatic impairment (Table 4). Circulating ANGPTL4 levels were also positively associated with HbA_1C_ and glucose levels in the category of impaired glycometabolism, and with AST, ALT and GGT in the category of hepatic impairment (Table 5). By contrast, circulating ANGPTL8 levels were positively associated with increased levels of LDL-cholesterol and TG and with decreased levels of HDL-cholesterol in the category of dyslipidemia, in addition to a positive association with obesity, as estimated by BMI (Table 6).

**Table 4:** Association between circulating ANGPTL3 levels and lab values relevant to hepatic impairment or inflammation (n = 800). A generalized linear model was used. To evaluate AST, ALT or GGT, covariates were adjusted by all variables listed in Table 3, minus the hepatic impairment category. To evaluate hs-CRP, covariates were adjusted by all variables listed in Table 3, minus the inflammation category. β, regression coefficient; 95% CI, 95% confidence interval; AST, aspartate transaminase; ALT, alanine transaminase; GGT, gamma-glutamyltransferase; and hs-CRP, high sensitivity C reactive protein.

	log(ANGPTL3)		
	β	95%CI	P
Hepatic impairment			
log(AST)	0.134	(0.035, 0.234)	0.008
log(ALT)	0.035	(-0.024, 0.095)	0.243
log(GGT)	0.444	(0.000, 0.089)	<.050
Inflammation			
log(hs-CRP)	0.041	(0.019, 0.064)	<.001

**Table 5:** Association between circulating ANGPTL4 levels and laboratory tests relevant to impaired glycometabolism, hepatic impairment or inflammation (n = 800). A generalized linear model was used. To evaluate HbA_1C_ or glucose, each covariate was adjusted by all variables listed in Table 3, minus the impaired glyometabolism category. To evaluate AST, ALT, or GGT, each covariate was adjusted by all variables listed in Table 3, minus the hepatic impairment category. To evaluate hs-CRP, each covariate was adjusted by all variables listed in Table 3, minus inflammation. β, regression coefficient; 95% CI, 95% confidence interval; HbA_1C_, hemoglobin A_1C_; AST, aspartate transaminase; ALT, alanine transaminase; GGT, Gamma-glutamyltransferase; and hs-CRP, high sensitivity C reactive protein.

	log(ANGPTL4)		
	β	95%CI	P
Impaired glycometabolism			
log(HbA1c)	0.463	(0.130, 0.797)	0.007
log(Glucose)	0.271	(0.044, 0.498)	0.019
Hepatic impairment			
log(AST)	0.236	(0.129, 0.343)	<.001
log(ALT)	0.077	(0.013 0.141)	0.018
log(GGT)	0.065	(0.018, 0.113)	0.007
Inflammation			
log(hs-CRP)	-0.021	(-0.045, -0.004)	0.094

**Table 6:** Association between circulating ANGPTL8 levels and laboratory tests relevant to obesity, impaired glycometabolism, or dyslipidemia (n = 800). A generalized linear model was used. To evaluate BMI, each covariate was adjusted by all variables listed in Table 3, minus obesity. To evaluate HbA1C or glucose, each covariate was adjusted by all variables listed in Table 3, minus the impaired glycometabolism catergory. To evaluate HDL, LDL or triglyceride, each covariate was adjusted by all variables listed in Table 3, minus the dyslipidemia category. BMI, body mass index; HbA1C, hemoglobin A1C; HDL, high-density lipoprotein cholesterol; LDL, low-density lipoprotein cholesterol; TG, triglyceride; β, regression coefficient; and 95% CI, 95% confidence interval.

	log(ANGPTL8)		
	β	95%CI	P
Obesity			
BMI	0.027	(0.016, 0.038)	<.001
Impaired glycometabolism			
log(HbA1c)	-0.246	(-0.637, 0.145)	0.217
log(Glucose)	0.001	(-0.252, 0.280)	0.919
Dyslipidemia			
HDL	-0.002	(-0.005, -0.001)	0.013
LDL	0.002	(0.001, 0.003)	0.004
log(TG)	0.324	(0.262, 0.386)	<.001

Molecular cooperation of ANGPTL3 and ANGPTL8 is reportedly critical for ANGPTL8-induced LPL inhibition and subsequent increases in TG levels [9, 24]. Therefore, we asked whether interactions of circulating ANGPTL3 and 8 levels were associated with altered circulating TG levels. As anticipated, multiple regression analysis revealed that an statistical interaction of ANGPTL3 and ANGPTL8 [log(ANGPTL3)*log(ANGPTL8)] was positively associated with increases in circulating TG levels after adjustment for possible confounders; however, that effect was less than the effect of circulating ANGPTL8 levels alone (Table 7).

**Table 7:** Association between serum triglyceride levels and ANGPTL3, ANGPTL8 or interaction of ANGPTL3 and ANGPTL8 (ANGPTL3*ANGPTL8) (n = 988). A generalized linear model was used. The model was adjusted by age, sex, smoking, drinking, obesity, past history of impaired glycometabolism or diabetes, including current use of insulin or glucose-lowering agents, past history of dyslipidemia, including current use of lipid lowering agents, or past history of hepatic impairment. ANGPTL, angiopoietin-like protein; β, regression coefficient; and 95% CI, 95 percent confidence interval.

	log(TG)		
	β	95%CI	P
log(ANGPTL3)	-0.003	(-0.076, 0.082)	0.943
log(ANGPTL8)	0.373	(0.312, 0.435)	<.001
log(ANGPTL3)*log(ANGPTL8)	0.160	(0.014, 0.306)	0.032

## Discussion

In the current study, we asked whether associations exist between circulating ANGPTL3, ANGPTL4, and ANGPTL8 levels and general medical indexes, including CVD risk, in 988 Japanese persons aged 27–84 years who had undergone a routine medical check-up. We found that increased levels of circulating ANGPTL3 were positively associated with liver dysfunction and inflammation, elevated ANGPTL4 levels were positively associated with impaired glycometabolism and liver dysfunction and inversely correlated with inflammation, and increased ANGPTL8 levels were associated with obesity, impaired glycometabolism, and dyslipidemia. Thus, circulating ANGPTL3, 4, and 8 levels reflect some CVD risk factors.

### Association between circulating ANGPTL3 levels and medical variables

Our study indicated that circulating ANGPTL3 levels are positively correlated with inflammation, as estimated by hs-CRP values. Although inflammation plays crucial roles in progression of metabolic diseases such as obesity or impaired glycometabolism [25], a positive correlation between inflammation and circulating ANGPTL3 remained statistically significant after adjustment for variables including obesity, impaired glycometabolism, dyslipidemia and hypertension, suggesting that circulating ANGPTL3 is independently associated with inflammation. A case control study by Conroy *et al*. reported that serum ANGPTL3 levels increase in patients with dengue hemorrhagic fever, a viral inflammatory disease [26], supporting a link between circulating ANGPTL3 levels and inflammation. Mechanisms linking ANGPTL3 and inflammation remain unclear; however, using cultured human microvascular vein endothelial cells, Camenisch *et al*. previously reported that ANGPTL3 induces angiogenesis through αvβ3 integrin signaling, suggesting that ANGPTL3 may promote inflammation [27].

High circulating ANGPTL3 levels were also positively correlated with hepatic impairment, as estimated by AST and GGT levels, but not ALT levels. ANGPTL3 expression is restricted to hepatocytes, and ANGPTL3 is secreted from only those cells, suggesting that ANGPTL3 is a “hepatokine” [28]. AST and GGT are deviation enzymes from injured hepatocytes [29], suggesting increased levels of circulating ANGPTL3 reflect hepatic injury.

An observational study reports that human subjects harboring loss-of-function *ANGPTL3* mutations show decreased serum ANGPTL3 protein levels, as well as decreased TG, LDL-cholesterol, and HDL-cholesterol, as well as lower incidence of coronary artery disease [12]. These findings suggest that changes in circulating ANGPTL3 levels may reflect altered lipid metabolism. Here, however, we did not observe a positive correlation between circulating ANGPTL3 levels and dyslipidemia. The ELISA assay used here detects only the full-length form of ANGPTL3. A previous report showed that full-length ANGPTL3 does not inhibit LPL activity, and that ANGPTL3 cleavage is important for its activation [23]. Therefore, the full-length form of ANGPTL3 detected may not be associated with altered TG levels. Further analysis is necessary to investigate whether active forms of ANGPTL3 mediate changes in circulating TG levels. A more recent clinical study demonstrated that ANGPTL3 suppression using monoclonal antibodies or antisense-oligonucleotides decreased circulating levels of LDL-cholesterol and TG in human subjects [12, 14, 15], although the underlying mechanism was not clarified. These findings suggest that full-length ANGPTL3 is also inactive in terms of LDL-cholesterol metabolism and that the circulating full-length ANGPTL3 that we detected here does not alter cholesterol levels. Further analysis is necessary to determine which form of ANGPTL3 is associated with altered circulating cholesterol levels.

### Association of circulating ANGPTL4 levels with medical variables

We also found that circulating ANGPTL4 levels are positively correlated with serum glucose and HbA_1C_ in the category of impaired glycometabolism, a finding consistent with a previous report [30]. We also found that serum ANGPTL4 levels were positively associated with serum AST, ALT and GGT levels in the category of hepatic impairment. In contrast to liver-specific expression of ANGPTL3, ANGPTL4 is broadly expressed in tissues such as lung, liver, heart, white adipose tissue, and brown adipose tissue [8, 31]. Our data reported here suggest that elevation of serum ANGPTL4 may reflect damage to hepatocytes or bile duct cells and may serve as a marker of liver damage. Mandard *et al*. previously reported that mice overexpressing ANGPTL4 in adipose tissue show predisposition to liver steatosis, potentially reflecting an association of ANGPTL4 with liver dysfunction including steatosis [32].

We also found that serum ANGPTL4 levels are inversely correlated with inflammation. A previous report showed that ANGPTL4 has anti-inflammatory activity in mice by suppressing expression of inflammatory genes expressed in macrophages [33]. It is now of interest to determine if circulating ANGPTL4 levels reflect anti-inflammatory activities in humans.

In human subjects, loss-of-function *ANGPTL4* mutations promote decreases in levels of circulating TGs and increases in HDL-cholesterol [13], suggesting that circulating ANGPTL4 concentrations are associated with lipid metabolism. We, however, did not observe a positive correlation between circulating ANGPTL4 levels and serum lipids. The ANGPTL4 truncated N-terminal coiled-coil domain is reportedly more active than full length forms in inhibiting LPL function [34]. Since the ELISA used here detects that domain, total circulating levels of ANGPTL4 reported in the current study include both full-length ANGPTL4 and its N-terminal coiled-coil domain. Thus, further investigation is necessary to reveal which form of ANGPTL4 is associated with dyslipidemia.

### Association of circulating ANGPTL8 levels and medical outcomes

ANGPTL8 is an atypical ANGPTL with an N-terminal coiled-coil domain but lacking a C-terminal fibrinogen-like domain [8, 9]. However, like ANGPTL3 and 4, ANGPTL8 reportedly inhibits LPL activity, and in mouse its activity is associated with increased levels of blood TGs [9]. In human subjects, Quagliarni *et al*. reported that mutations that suppress ANGPTL8 activity are associated with decreased levels of circulating LDL- and HDL-cholesterol levels, but not with circulating TG levels, although transgenic mice overexpressing *Angptl8* in liver exhibited hypertriglycemia [9]. Interestingly, our current study reveals a positive correlation of circulating ANGPTL8 levels with both TG and LDL-cholesterol levels in circulation and an inverse correlation with serum HDL-cholesterol levels. Thus, circulating ANGPTL8 levels reflect dyslipidemia in human subjects.

Our study also reveals a positive correlation of circulating human ANGPTL8 levels with obesity, consistent with a previous study of human subjects [35]. In that study, interventions such as exercise training significantly decreased circulating ANGPTL8 levels in obese subjects, and had a lesser effect on ANGPTL8 levels in non-obese subjects [35]. Interestingly, ANGPTL8 is expressed in both white and brown adipose tissue in addition to liver [8]. Moreover, *ANGPTL8* expression increases during differentiation of primary cultured human or mouse pre-adipocytes to adipocytes [36]. Taken together with these findings, ANGPTL8 secreted from adipocytes may underlie increases in circulating ANGPTL8 levels seen in obesity.

Some reports suggest that circulating ANGPTL8 levels are positively correlated with circulating levels of glucose or HbA1c [37, 38]. However, we did not observe a significant correlation between circulating ANGPTL8 levels and HbA1c and or glucose levels in the category of impaired glycometabolism. Further investigation is required to assess whether circulating ANGPTL8 levels reflect pathology of diabetes.

Reports also suggest that circulating levels of ANGPTL3, 4, and 8 reflect various health concerns including CVD risk, although these reports vary [16–21, 30, 35]. These differences may reflect backgrounds of study subjects, including age, sex, race, and health status, or differences in ELISA sensitivity or specificity. In current study, we focused on a population undergoing routine medical checkups and measured circulating levels of 1) full length ANGPTL3, 2) total ANGPTL4 including full-length and N-terminal coiled-coil domain, and 3) full length ANGPTL8. Our results partially support previous reports. Further investigations are needed to validate relationships between circulating ANGPTL levels and human medical status by focusing on differences in study population or technical disparities attributable to ELISA kits.

Our current study has some limitations. First, circulating ANGPTL levels reported here differ from those reported in other reports [16, 20, 26, 30]. These differences may be due to differences of ELISA kits, including sensitivity, specificity, form of ANGPTL protein detected, antibody cross-reactivity, or differences in protein standards. These differences are unavoidable, as to date, there are no internationally-accepted standards for measuring circulating ANGPTLs levels. Second, given that serum specimens assayed here were thawed twice before analysis, the freeze-thaw protocol may slightly alter concentration of serum ANGPTLs.

In summary, the current study reveals that levels of circulating ANGPTL3, 4, and 8 reflect some risk factors of CVD development. In particular, we found that circulating human ANGPTL8 levels were positively correlated with obesity and dyslipidemia, as estimated by increased TG and LDL-cholesterol levels and decreased HDL-cholesterol levels, suggesting that circulating ANGPTL8 concentrations predict future CVD development. However, the cross-sectional study design used here limits our interpretation of whether there is an association between ANGPTL8 levels and future CVD events. Further prospective cohort studies will be required to answer this question.
